# Vitamin D and oestrogen receptor polymorphisms in developmental dysplasia of the hip and primary protrusio acetabuli – A preliminary study

**DOI:** 10.1186/1477-5751-6-7

**Published:** 2007-06-28

**Authors:** Birender Kapoor, Colin Dunlop, Charles Wynn-Jones, Anthony A Fryer, Richard C Strange, Nicola Maffulli

**Affiliations:** 1 Department of Trauma and Orthopaedic Surgery, Arrowe Park Hospital, Arrowe Park Road, Upton, Wirral. CH49 5PE, UK; 2 Department of Trauma and Orthopaedic Surgery, Ninewells Hospital, Dundee. DD1 9SY, UK; 3 Department of Trauma and Orthopaedic Surgery, Keele University School of Medicine, University Hospital of North Staffordshire, Thornburrow Drive, Hartshill, Stoke on Trent, Staffordshire, ST4 7QB, UK; 4 Human Genomics Research Group, Institute for Science and Technology in Medicine, Keele University School of Medicine, University Hospital of North Staffordshire, Thornburrow Drive, Hartshill, Stoke on Trent, Staffordshire, ST4 7QB, UK

## Abstract

We investigated the association of developmental dysplasia of the hip (DDH) and primary protrusion acetabuli (PPA) with Vitamin D receptor polymorphisms Taq I and Fok I and oestrogen receptor polymorphisms Pvu II and Xba I. 45 patients with DDH and 20 patients with PPA were included in the study. Healthy controls (n = 101) aged 18–60 years were recruited from the same geographical area. The control subjects had a normal acetabular morphology based on a recent pelvic radiograph performed for an unrelated cause. DNA was obtained from all the subjects from peripheral blood. Genotype frequencies were compared in the three groups. The relationship between the genotype and morphology of the hip joint, severity of the disease, age at onset of disease and gender were examined. The oestrogen receptor Xba I wild-type genotype (XX, compared with Xx and xx combined) was more common in the DDH group (55.8%) than controls (37.9%), though this just failed to achieve statistical significance (p = 0.053, odds ratio = 2.1, 95% CI = 0.9–4.6). In the DDH group, homozygosity for the mutant Taq I Vitamin D receptor *t *allele was associated with higher acetabular index (Mann-Whitney U-test, p = 0.03). Pvu II pp oestrogen receptor genotype was associated with low centre edge angle (p = 0.07). This study suggests a possible correlation between gene polymorphism in the oestrogen and vitamin D receptors and susceptibility to, and severity of DDH. The Taq I vitamin D receptor polymorphisms may be associated with abnormal acetabular morphology leading to DDH while the Xba I oestrogen receptor XX genotype may be associated with increased risk of developing DDH. No such correlations were found in the group with PPA.

## Background

Developmental Dysplasia of the hip (DDH) and Primary Protrusio Acetabuli (PPA) encompass the spectrum of acetabular development from a shallow acetabulum in DDH to a deep acetabulum in PPA. Both have, as yet, an indeterminate aetiology, variable clinical presentation, and result in early onset osteoarthritis of the hip [1]. A genetic aetiology has been proposed in DDH [2], while the aetiology of PPA is widely debated. Eppinger believed that PPA results from a failure of normal ossification of the tri-radiate cartilage [3]. The possible genetic nature of transmission of this disorder was noted by D'Arcy *et al *[4]. Idiopathic PPA may represent a hitherto unidentified metabolic defect.

Hypothetically, it may be conceived that small numbers of genomic polymorphisms may well affect acetabular morphology and capsular laxity, and provide a spectrum of morphology across the population with the major dysmorphism leading to clinical apparent disease.

Genetic variation in hormone-related genes may represent a possible significant determinant of risk or severity, especially when considering the proposed effect of joint laxity on DDH [5,6]. The human ESR1 gene is located on chromosome 6q25. It comprises eight exons separated by seven intronic regions and spans more than 140 kilobases. The most widely studied polymorphic regions are the Pvu II and Xba I restriction fragment length polymorphisms in intron 1 and the (TA) *n *variable number of tandem repeats (VNTR) within the promoter region of the gene. The ESR1 is a ligand-activated transcription factor composed of several domains important for hormone binding, DNA binding, and activation of transcription. Alternative splicing results in several ESR1 mRNA transcripts, which differ primarily in their 5' untranslated regions.

The VDR gene is located on chromosome 12q12–q14. It contains 11 exons and spans approximately 75 kb. The Fok I site polymorphism is present in exon 2 of the VDR gene whereas Taq 1 resides in exon 9. The VDR gene is associated with osteoporosis [7], osteoarthritis [8,9], and prostate cancer [10]. The ESR1 gene has been linked with osteoarthritis [11], osteoporosis [12-16], breast cancer [17], and testicular cancer [18]. Both genes are involved in bone metabolism and development.

The VDR and the ESR1 genes are interesting because they encode proteins that are important transcription factors as key players in the respective signal transduction pathways. Several interactions between the vitamin D and oestrogen endocrine system have been described. 1,25-Dihydroxyvitamin D_3 _(1,25-(OH)_2_D_3_) and 17β-estradiol (E_2_) have a mutual effect on their biosynthesis [19,20] and receptor expression [21]. Also, some genetic studies found an interaction between ESR1 and VDR genotypes with respect to bone density [22]. Suarez *et al*. found an interactive effect of ESR1 and VDR gene polymorphisms on growth in infants [23]. Although oestrogen receptor polymorphisms have been studied in relation to DDH [24], there has not yet been a definite evidence of their role in causation. Also, to our knowledge this approach has not been used in the study of PPA. We therefore performed a study to identify the possible association between genetic polymorphism at these loci and the presence of DDH and PPA. We also explored the effect of genotype on acetabular morphology and severity of the conditions.

## Patients and methods

We recruited 45 patient with DDH and 20 patients with PPA. In all patients, a diagnosis of DDH or PPA was made on the basis of clinical and radiographic examination. A control group of 101 subjects (age 18–60) was recruited from the same geographical region and the same ethnic group from the hospital radiology database. These subjects had normal hip joints on the basis of recently performed pelvic radiographs for unrelated causes. All patients and control subjects were Caucasian. The demographic data on the study population is shown in Table 1. All participants in this investigation were interviewed and examined to obtain clinical history, family history, and a peripheral blood sample through venipuncture. Informed written consent was obtained from all the subjects prior to their participation in the study. Ethics approval was obtained from the Local Research Ethics Committee.

**Table 1:** Demographic details of groups studied

	**Control**	**DDH**	**PPA**
**Number**	101	45	20
**Age – Mean (range)**	42.6 (19–60)	46.1 (22–69)	47.5 (28–65)
**Age at presentation – Mean (range)**	NA	14.5 (0–48)	22.05 (5–46)
**Gender**	47 M/54 F	4 M/41 F	3 M/17 F
**Positive Family History**	NA	3	0

NA = not applicable

### Radiographic measurements

Pelvic radiographs were obtained, and radiographical variables (acetabular index and centre edge angle) of the hip joint were measured by a single individual using a uniform technique (BK). The researcher had been specially trained in radiographic measurement techniques, and reached an intra-observer variation of less than two degrees.

The centre-edge angle was measured as described by Wiberg, between a vertical line drawn from the centre of the femoral head to a line drawn from the centre of the head to the lateral edge of the acetabulum on antero-posterior radiographs of the pelvis [25]. The acetabular index was measured as described by Sharp, between the inter-tear drop line and the weight bearing dome [26]. The radiographic data of the study population are presented in Table 2.

**Table 2:** Radiographic variables of the groups studied

**Radiographic variable**	**Control Mean (range)**	**DDH Mean (range)**	**PPA Mean (range)**
**Centre edge angle (left)**	34.3 (32.9–35.6)	20.1 (15.4–23.1)	45.1 (35.7–46.0)
**Centre edge angle (right)**	34 (32.8–35.2)	24 (20.2–28.5)	42.9 (37.9–46.6)
**Acetabular index (left)**	9.9 (9.2–10.6)	27.4 (24.7–30.5)	-4.6 (-7.9–-0.6)
**Acetabular index (right)**	10.2 (9.4–10.9)	20.3 (16.3–24.2)	-3.6 (-7.0–0.5)

### Genotype assays

All genotype assays were performed at the Human Genomics Research Group, University Hospital of North Staffordshire by two individuals (BK, CD), and the results validated by an independent, blinded observer examining the agarose gels (AF). 10% of the assays were repeated and analysed by an independent observer. The PCR assays were performed with at least one known DNA genotype (positive control), one negative control (no DNA), and known molecular weight markers. At least 15% of the samples were re-assayed, and the relevant genotype confirmed. DNA was extracted from the peripheral blood samples collected into EDTA using the phenol-chloroform extraction method. PCR RFLP-based assays were performed to identify alleles containing Xba I and Pvu II polymorphisms on the oestrogen receptor 1 (ESR1). Taq I and Fok I polymorphisms on the vitamin D receptor gene or VDR were also analysed. The PCR products were then digested with their respective restriction enzymes and were examined after electrophoresis on 2% agarose gels. The wild-type X, P, T, F and mutant x, p, t and f alleles were identified by the expected fragment sizes following restriction enzyme digestion.

### Statistical analysis

Data were analysed using Stata software (version 8, StataCorp, Texas, US). Differences in genotype frequencies of the ESR1 (Xba I, Pvu II) and VDR (Taq I, Fok I) polymorphisms were examined in the three groups using chi-square tests. Association of genotypes with acetabular index and centre edge angles was performed using the Mann-Whitney U test.

## Results

Table 3 shows the genotype distribution for the four polymorphic sites. The oestrogen receptor Xba I wild-type genotype (XX, compared with Xx and xx combined) was more common in the DDH group (55.8%) than controls (37.9%), though this failed to achieve statistical significance after Bonferroni correction (p = 0.106). Similarly, the VDR Fok I ff genotype (compared with FF and Ff combined) was more common in DDH patients than controls but was not statistically significant (p = 0.18 after Bonferroni correction). No other significant associations were identified.

**Table 3:** Genotype frequency

**ESR Xba I**	**CONTROL**	**DDH**	**PPA**
**XX**	33(37.9%)	24(55.8%)	6(30%)
**Xx**	45(51.7%)	18(41.9%)	12(60%)
**xx**	9(10.3)	1(2.3%)	2(10%)
			
**ESR Pvu II**			
**PP**	14(15.2%)	4(8.9%)	5(25%)
**Pp**	47(51.1%)	27(60%)	11(55%)
**pp**	31(33.7%)	14(31.1%)	4(20%)
			
**VDR Fok I**			
**FF**	33(34.4%)	15(33.3%)	8(42.1%)
**Ff**	52(54.2%)	20(44.4%)	9(47.4%)
**ff**	11(11.5%)	10(22.2%)	2(10.5%)
			
**VDR Taq I**			
**TT**	31(34.1%)	18(41.9%)	7(43.8%)
**Tt**	46(50.5%)	20(46.5%)	5(31.3%)
**tt**	14(15.4%)	5(11.6%)	4(25%)

The most relevant radiographic variables of both these conditions (i.e. centre edge angle and acetabular index) on the affected and non-affected sides were also compared with genotype. In the DDH group, homozygosity for the Taq I Vitamin D receptor *t *allele was associated with higher acetabular index on the affected side This was demonstrated using the Mann-Whitney U-test. However, this again was not statistically significant following Bonferroni correction. (p = 0.06). In this group, the Pvu II pp oestrogen receptor genotype was associated with low centre edge angle on the affected side though this did not achieve statistical significance (p = 0.14). No other significant or near-significant associations were identified.

## Discussion

Developmental dysplasia of the hip and primary protrusio acetabuli are two common developmental disorders of the hip joint, with significant associated morbidity. Efforts have been made for the last 40 years for early identification of developmental hip dysplasia, as early correction of anatomy can produce a hip which has greater chances will last to late adulthood without major reconstructive surgery [27]. If detected early, secondary osteoarthritis can be partially prevented or at least delayed. Currently, screening for DDH in the UK is performed by clinical examination and ultrasound scanning of patients at risk. Blanket ultrasound screening has been proposed, but is not significantly better than at risk or selective screening[28-30]. Therefore, it would be highly desirable to identify predictors to a high risk population.

Aetiological factors for both DDH and PPA are obscure. There are definite pointers towards a genetic basis, but no concrete evidence to support it. Some recent studies have investigated the genetic basis of DDH. Granchi et al found an association between osteoarthritis secondary to developmental hip dysplasia and vitamin D receptor polymorphism Bsm I. To our knowledge, there are no similar studies performed for PPA. PPA and DDH may actually represent two ends of a spectrum in the phenotypic outcome of a genotypic variation. This formed the basis of studying same gene polymorphisms for these two conditions.

A detailed study of 589 index patients and 1897 first degree relatives in the 1960's established a familial transmission in non-syndromic hip dysplasia [31]. There was significant shallowing of the acetabulum in parents of children with DDH, and a higher proportion of children with DDH and their first degree relatives were lax-jointed. Based on this study, Wynne-Davis proposed two different gene systems, one affecting joint laxity and the other affecting the shape of acetabulum to be responsible for the causation of DDH. Carter and Wilkinson reported increased incidence of joint laxity with DDH in 1964 [32]. With the recent advances in genetic techniques, there has been a renewed interest to explore the inheritance of this disorder. Solazzo et al performed a complex segregational analysis on 171 pedigrees collected through probands affected by non-syndromic DDH, and reiterate a two locus theory [33], against the previous hypothesis that disease inheritance in familial non-syndromic DDH is polygenic.

The oestrogen receptor Xba I wild-type genotype (XX, compared with Xx and xx combined) was more common in the DDH group (55.8%) than controls (37.9%). Though this failed to achieve significance, it may warrant further investigation. In the DDH group, homozygosity for the mutant Taq I Vitamin D receptor *t *allele was associated with higher acetabular index. This may represent an important aetiological association with DDH. Similarly, the Pvu II oestrogen receptor was associated with a low centre edge angle. Though this did not reach significance it may represent an association with severity of DDH. Our results are indicative, rather than conclusive, of the association between developmental dysplasia of the hip and oestrogen and vitamin D receptor polymorphisms in the studied population groups. Our study population was kept homogenous, and is representative of Caucasian population in a well defined region of the UK. This was also disadvantageous in the fact that that the total number of patients recruited was low. However, the total number of cases does compare well with other recent genetic studies on DDH. We are aware of much larger case series of patients with DDH from tertiary referral centres. However, these have not been characterised in genetic studies. Indeed, population homogeneity would be an issue in larger series due to the current known prevalence of DDH and PPA in our population.

The present study has shown possible genetic associations between DDH and vitamin D and oestrogen receptor polymorphisms. Further work with a larger series of patients and possibly more candidate gene polymorphisms may well shed more light on these associations. We hope that the genetic associations identified in the present study may lead to more accurate means to identify at risk populations. The associations, whether positive or negative may help us to understand the mode of transmission of this condition.

## Competing interests

We hereby categorically declare that none of the authors have any financial or non-financial competing interests in the publishing of this manuscript.

## Authors' contributions

BK and CD performed the molecular assays. CD and CHWJ were involved in the conceptualisation of the study as well as defining the radiographic measurement methods. RCS and AAF were involved in set up and operational help with the genetic assays. NM planned the study, supervised BK and CD, was instrumental in drafting and revising the manuscript, and give final approval for the publishing of this document. All the authors have read and approved the final manuscript.
